# Luteolin Binds Streptolysin O Toxin and Inhibits Its Hemolytic Effects and Cytotoxicity

**DOI:** 10.3389/fphar.2022.942180

**Published:** 2022-07-07

**Authors:** Tingting Guo, Peng Liu, Zeyu Wang, Yuling Zheng, Wenhua Huang, Decong Kong, Lizhong Ding, Qingyu Lv, Zhongtian Wang, Hua Jiang, Yongqiang Jiang, Liping Sun

**Affiliations:** ^1^ College of Chinese Medicine, Changchun University of Chinese Medicine, Changchun, China; ^2^ State Key Laboratory of Pathogen and Biosecurity, Beijing Institute of Microbiology and Epidemiology, Academy of Military Medical Sciences (AMMS), Beijing, China; ^3^ Affiliated Hospital to Changchun University of Chinese Medicine, Changchun, China

**Keywords:** Group A *streptococcus*, streptolysin O, luteolin, toxin, hemolysis

## Abstract

Group A *streptococcus* (GAS, *Streptococcus pyogenes*) is a common pathogen that can cause a variety of human diseases. Streptolysin O (SLO) is an exotoxin produced by GAS. It is a pore-forming toxin (PFT) that exhibits high *in vivo* toxicity. SLO enables GAS to evade phagocytosis and clearance by neutrophils, induces eukaryotic cell lysis, and activates inflammatory bodies. Luteolin is a natural compound that is produced by a wide range of plant species, and recent studies have shown that luteolin can inhibit the growth and alter the morphological of GAS. Here, we reported that luteolin can weaken the cytotoxicity and hemolytic activity of SLO *in vitro*. Briefly, luteolin bound SLO with high affinity, inhibited its dissolution of erythrocytes, affected its conformational stability and inhibited the formation of oligomers. To further verify the protective effect of luteolin, we used an *in vitro* SLO-induced human laryngeal carcinoma epithelial type-2 cells (HEp-2) model. Notably, our results showed luteolin protected HEp-2 cells from SLO induced cytotoxicity and changed in cell membrane permeability. In addition, we explored the role of luteolin in protecting mice from GAS-mediated injury using an aerosolized lung delivery model, and our results indicate that luteolin increases murine survival rate following inoculation with a lethal dose of GAS, and that survival was also associated with decreased pathological damage to lung tissue. Our results suggest that luteolin may be a novel drug candidate for the treatment of GAS infection.

## Introduction

GAS is a Gram-positive bacterium that causes a variety of diseases ranging from pharyngitis, erysipelas, and cellulitis to more severe, life-threatening diseases such as streptococcal toxic shock syndrome and necrotizing fasciitis. Additionally, GAS infection can also cause severe immune-mediated diseases such as acute glomerulonephritis, rheumatic fever, and rheumatic heart disease (Martin and Green, 2006; Steer et al., 2007; Walker et al., 2014). Current research reports that nearly 5,00,000 people die of GAS infection annually (Nelson et al., 2016).

As a human adapted pathogen, GAS is protected by a number of cell surface-bound and secreted virulence factors that contribute to its versatility as a pathogen and help it to subvert host innate immune defenses including phagocytosis, complement deposition, antibody opsonization, antimicrobial peptides, and neutrophil killing mechanisms (Mitchell, 2003; Walker et al., 2014). Among them, Streptolysin O (SLO), an extracellular product produced by almost all GAS strains that belongs to the cholesterol-dependent cytolysin (CDC) proteins, a conserved family of β-barrel PFTs, is one of the most important virulence factors in the GAS arsenal (Shewell et al., 2014; Lukoyanova et al., 2016). PFTs are one type of important virulence factors including α-Hemolysin (Hla), Pneumolysin O (PLY), Listeriolysin O (LLO), and Suilysin (SLY), etc. (Los et al., 2013; Dal Peraro and van der Goot, 2016). The SLO monomer binds to the host cell membrane through the conserved region of the C-terminal domain 4 (D4), then forms a protein multimer to create a barrel structure that enables it to penetrate the host cell, resulting in cell lysis and subsequent organ system dysfunction during invasive infections (Tweten, 2005; Rossjohn et al., 2007; Mozola and Caparon, 2015; van Pee et al., 2017). Additionally, during GAS infection SLO mediates the transfer of NAD^+^ glycohydrolase SPN into the host cell cytosolic compartment, in which it exerts its toxic effects (Bricker et al., 2002). Studies have shown that SLO plays a key role in related disease phenotypes and outcomes caused by GAS infection, therefore, specific anti-toxin treatments are expected to impact the clinical outcome (Clarke et al., 2021). Thus, screening for inhibitors of SLO would facilitate the development of anti-SLO treatments for infections.

With the increasing resistance of bacteria to antibiotics, more and more researchers are developing new antibacterial drugs by targeting virulence factors such as hemolysin (Jiang et al., 2019; Lu et al., 2019; Vita et al., 2020). Chinese herbal medicines are rich in resources and have various components. Starting from a foundation of Chinese herbal medicines and then focusing on their active ingredients may have broad prospects in the development of new antibacterial drugs against bacterial virulence factors. In clinical treatment of tonsillitis and other diseases caused by GAS-infection, Traditional Chinese Medicines with “clear away heat and toxic material” are often used, such as *Tanacetum parthenium*, *Trollius chinensis*, *Taraxacum officinale*, *Scutellaria barbata*, *Schizonepeta tenuifolia*, *Satureja parvifolia*, *Platycodon grandiflorum*, etc. These medicines all contain flavonoid compounds. Flavonoids, also known as polyphenols, have been widely studied because of their good bacteriostasis, including the inhibition of multi-drug resistant bacteria (Biharee et al., 2020). Luteolin is a member of the flavonoid family of natural compounds, which are commonly found in many types of plants used for medicine and food such as natural herbal drugs, vegetables, fruits, luteolin-rich herbal extracts have been widely used as new traditional herbal medicines and possesses numerous biological effects including anti-inflammatory, anti-oxidant, anti-hypertensive, anti-cancer activities (Lin et al., 2008; Lopez-Lazaro, 2009). Additionally, luteolin has been proved to have antibacterial activity against many types of bacteria (Lv et al., 2009; Siriwong et al., 2015; Guo et al., 2020) and can eliminate antibiotic resistance in multidrug-resistant *Trueperella pyogenes* (Zhang et al., 2019). Recent studies indicate that luteolin can inhibit the formation and apoptosis of macrophage foam cells, can suppress activation of the NLRP3 inflammatory corpuscle and promote polarization of macrophages towards the M2 phenotype (Chen et al., 2014; Zhang et al., 2016).

As mentioned earlier, the SLO protein is a key virulence factor of GAS and plays an important role in its infection process. Therefore, we tested the hypothesis that luteolin may be an effective compound against SLO protein-related functional activities. Here, we showed that luteolin binds SLO with high affinity through a non-conventional binding site located in domains 1 and 3. This binding activity inhibits SLO cytotoxicity *in vitro* in HEp-2 and also significantly reduces its hemolytic effects in red blood cells (RBCs). These results support the hypothesis that luteolin protects cells from SLO toxicogenic effects. In addition, we also verified the anti-infection effect of luteolin *in vivo* through a mouse infection model.

## Materials and Methods

### Bacterial Strain, Cell and Culture Conditions

Group A *Streptococcus* (ATCC 700294) was selected for use in this study. The selected GAS strain was cultured at 37°C in Todd-Hewitt broth amended with 0.2% yeast extract (THY) (BD, United States). Human laryngeal carcinoma epithelial type-2 cells (HEp-2) were purchased from ATCC and cultured in Dulbecco’s Modified Eagle Medium (DMEM) (Sigma, United States), supplemented with 10% fetal bovine serum (FBS) (Gibco, United States), and 100 μg/ml of penicillin and streptomycin (Solarbio, China). Cells were cultured at 37°C under 5% CO_2_ and counted using the Invitrogen Countess II chamber (Thermo Fisher, United States).

Luteolin (purity ≥ 98%) was purchased from Shanghai yuan ye Bio-Technology Co., Ltd. (Shanghai, China) and dissolved in distilled water to make stock solutions of various concentrations. The luteolin solution was filter sterilized through a 0.22 μm microfiltration membrane.

### Construction, Expression, and Purification of Proteins

Primers were designed to amplify the GAS gene encoding SLO using DNA extracted from GAS strain M1 as the template; the primers used are listed in Table 1. *BamH* I and *Sac* I restriction enzymes were used to digest pET28a prokaryotic expression vector and the amplified SLO gene, were then cloned into the vector using a Gibson assembly strategy (NEB). To express the recombinant protein, the pET28a-SLO vector was transformed into *Escherichia coli* BL21 (DE3). PLY, LLO, SLY were constructed in the same way (strains used were *Streptococcus pneumoniae* TIGR4, *Listeria monocytogenes* 10403S, and *Streptococcus suis* 05ZYH33; primers are listed in Table 1). The cells were cultured at 37°C until absorbance at OD_600_ reached 0.6–0.8, SLO, PLY, LLO expression was induced with 0.5 mM IPTG, and cells were harvested after growing for an additional 4 h at 25°C or overnight at 16°C. SLY was induced with 1 mM IPTG and harvested after growing 4 h at 28°C. The harvested cells were resuspended in washing buffer consisting of 20 mM PB, 20 mM imidazole, and 0.5 M NaCl (pH 7.4), and lysed by sonication. The cell lysate was centrifuged at 8,000 × *g* for 10 min, and the supernatant was loaded onto a HisTrap HP column. The recombinant protein was bound to a Nickel-affinity chromatography column, and the column was flushed with wash buffer consisting of 20 mM PB, 0.5 M imidazole, and 0.5 M NaCl (pH 7.4). The His-tagged protein was eluted in elution buffer containing 20 mM PB, 0.5 M imidazole and 0.5 M NaCl (pH 7.4). The purified product was identified by sodium dodecyl sulfate polyacrylamide gel electrophoresis (SDS-PAGE).

**TABLE 1:** Primers used in this study.

Primer	Sequence (5′–3′)
*slo* forward	TGG​ACA​GCA​AAT​GGG​TCG​CGG​ATC​CCT​TGC​TCC​CAA​AGA​AAT​GCC
*slo* reverse	GCA​AGC​TTG​TCG​ACG​GAG​CTC​CTA​CTT​ATA​AGT​AAT​CGA​ACC​ATA​TG
*ply* forward	TGG​ACA​GCA​AAT​GGG​TCG​CGG​ATC​CAT​GGC​AAA​TAA​AGC​AGT​AAA​TGA​C
*ply* reverse	GCA​AGC​TTG​TCG​ACG​GAG​CTC​CTA​GTC​ATT​TTC​TAC​CTT​ATC​TTC​TA
*llo* forward	TGG​ACA​GCA​AAT​GGG​TCG​CGG​ATC​CGA​TGC​ATC​TGC​ATT​CAA​TAA​AG
*llo* reverse	GCA​AGC​TTG​TCG​ACG​GAG​CTC​TTA​TTC​GAT​TGG​ATT​ATC​TAC​TTT​ATT​AC
*sly* forward	TGG​ACA​GCA​AAT​GGG​TCG​CGG​ATC​CGA​TTC​CAA​ACA​AGA​TAT​TAA​TCA​G
*sly* reverse	GCA​AGC​TTG​TCG​ACG​GAG​CTC​TTA​CTC​TAT​CAC​CTC​ATC​C

### Hemolytic Activity Assay

RBCs hemolysis assay was modified with reference to previous protocols (Dong et al., 2018; Dong et al., 2021). Briefly, SLO (0.1 μg/ml) was added to the solution with or without luteolin; Sheep RBCs were harvested and added to the solution to a final concentration of 2% (v/v). The whole mixture was incubated for 30 min at 37°C and centrifuged at 5,000 rpm to obtain the OD_540_ of the supernatants. Samples treated with 0.1% Triton-X 100 were fully hemolytic and used as positive controls. The final hemolysis ratio was expressed as [OD_54_0(samples)/OD540(positive controls)] × 100%. In addition, the hemolytic activity of SLY, PLY, and LLO proteins was measured by the same way. To evaluate the effect of luteolin on the hemolytic activity of natural SLO protein, GAS culture supernatant was harvested with centrifugation at 10,000 rpm for 5 min. Subsequently, 10% (v/v) GAS culture supernatant was added to the hemolysis buffer with or without luteolin for 30 min prior to detection, and the hemolysis efficiency was determined using the same method performed with SLO protein.

### Molecular Docking

The small molecule ligand was set to be flexible, and the protein receptors were rigid. Luteolin was docked into SLO (PDB code: 4hsc), SLY (PDB code: 3hvn), PLY (PDB code: 5cr6), and LLO (PDB code: 4cdb) 3D X-ray structures, using the docking program AutoDock 4.0 (AutoDock program, The Scripps Research Institute, La Jolla, CA) (Morris et al., 2009; Hu et al., 2010). PyMOL and Ligplus programs were used to visually analyze the docking conformation.

### Molecular Dynamics Simulation and Calculation of the Binding Free Energy.

A Molecular dynamics simulation (MD simulation) of the SLO-luteolin complex was carried out using the Gromacs 5.0.4 package to explore the binding mode of the complex. The protein used ff14SB force field parameters, the small molecule ligand used gaff general force field parameters, and the atomic charge of AM1-BCC was calculated by the ANTECHAMBER module. The binding free energy of the complex was calculated by the MMPBSA.py module (Jogalekar et al., 2010; Rungrotmongkol et al., 2010; Vorontsov and Miyashita, 2011).

### Surface Plasmon Resonance Analysis

The affinity and kinetics of luteolin to SLO, SLY, PLY, LLO were measured by surface plasmon resonance (SPR) at 25°C on a REICHERT 4SPR (Reichert, United States) using carboxymethyl dextran hydrogel surface sensor chips. Proteins were dissolved in 10 mM sodium acetate (pH 4.5) and immobilized on the chip with 6,000 response units (RU) at a flow rate of 10 μl/min. Luteolin was serially diluted in PBST buffer (PBS containing 0.005% Tween 20) to concentrations of 0.125, 0.25, 0.5, 1, 2, and 4 μg/ml. Each of the six concentrations was injected at a flow rate of 25 μl/min for 3 min; for dissociation, the flow rate was set at 25 μl/min for 5 min. To regenerate channels, 10 mM glucopyranoside (Sigma-Aldrich St. Louis, MO, United States) was injected for 90 s at a flow rate of 25 μl/min, followed by injection of PBST buffer for 15 s until the RU reached the original reading. All injections were performed at 25°C. Reference cell values and signal from buffer injection controls were subtracted, and the sensorgram traces were fitted to a 1:1 Langmuir binding model using the data analysis program Scrubber 2.0 (BioLogic Software, Canberra, Australia) to calculate ka, kd, and KD values. The figures were made using Prism (GraphPad Software, Inc.).

### Determination of Protein Secondary Structure

MMS measurements were conducted using the automated AQS3 Pro system (RedshiftBio) with AQS3 analytics software. Solutions of SLO protein and protein + luteolin, each at 0.8 mg/ml, were prepared in PBS. All samples and their corresponding buffer blanks were preloaded into a 24-well plate in a pairwise manner. The samples and buffers were degassed using a built-in well plate degasser for 30 min. An automated testing protocol, including all reference buffer and sample measurements, was set up in the acquisition software in triplicate for each experiment.

### Determination of Melting and Aggregation Temperatures

Thermal and colloid stabilities of SLO proteins before and after luteolin treatment were evaluated using the Uncle/UNit system (Unchained Labs, Pleasanton, CA, United States). The barycentric mean (BCM) and static light scattering (SLS) at 266 nm were used to monitor the evolution of protein structures in response to increasing the temperature from 25°C to 95°C at a rate of 0.5°C/min. The label-free fluorescence from intrinsic aromatic amino acid residues excited by the inset excitation wavelength of 266 nm was collected for melting temperature (the midpoint of unfolding event, Tm) calculation, while SLS at 266 nm was monitored, respectively, to determine the temperature where SLO protein starts to aggregate (the starting point of aggregation event, Tagg).

### Oligomerization Analysis

Purified SLO (1 mg/ml) incubated with different concentrations of luteolin was incubated at 37°C for 30 min. Purified SLO without the luteolin was used as a control. An equal volume of each sample was incubated at 40°C for 10 min in 1 × SDS-PAGE loading buffer without β-mercaptoethanol (β-ME). Equal quantities of bacterial protein were separated by SDS-PAGE and transferred onto a polyvinylidene fluoride (PVDF) membrane. The membrane was incubated with rabbit polyclonal antibody raised against Streptolysin O (1:500; Abcam, United Kingdom), and probed with Goat anti-Rabbit IRDye®680RD (1:5,000, LI-COR, United States). The protein bands were visualized using an Odyssey SA (LI-COR, United States).

### Cell Viability Assay and Protection Experiment

To test the protection effect of luteolin, a cell culture model of HEp-2 cells were established. And first, the effect of luteolin on the viability of HEp-2 cells was determined by a cell counting kit-8 (CCK-8) assay according to the manufacturer’s description (MCE, United States). Cells were seeded at a density of 1 × 10^4^ into 96-well plates with or without different concentrations of luteolin, and DMEM alone and the CCK-8 reagent system, with or without cells, were used as the control group and blank group, respectively. After incubation at 37°C for 24 h, 10 μl of CCK-8 solution was added to each well and cells incubated for 3 h more, after which the absorbance at OD_450_ was measured using a multifunctional enzyme labeling instrument (Thermo Fisher, United States).

For determination of IC50 values, dose-response (1, 2, 4, 8, and 16 μg/ml SLO) and time-course (0.5, 3, and 6 h) experiments were performed. Briefly, HEp-2 cells were seeded in flat-bottomed 96-well plates at a concentration of 1 × 10^4^ cells/well. After 24 h, confluent cell monolayers were placed in serum-free medium and treated with increasing concentrations of SLO. To evaluate the effect of luteolin, HEp-2 cells were treated with 3 μg/ml of SLO for 6 h, in the absence or presence of luteolin (2, 4, 8, and 16 μg/ml). As above, cell viability was measured with CCK-8 solution to evaluate the protective effect of luteolin on cells.

### Cell Labeling With Fluorescent Dye

HEp-2 cells were seeded in 8-well Lab-Tek chambered cover glass plates (Thermo Fisher Scientific, United States) at a concentration of 5 × 10^4^ cells/well. After 24 h, cells were placed in serum-free medium and treated with 3 μg/ml SLO with or without luteolin (8 μg/ml). After 6 h, cells were stained with the LIVE/DEAD™ Cell imaging Kit (Thermo Fisher Scientific, United States) following the manufacturer’s instructions. Cells were immediately analyzed and acquired using the FV1000 confocal microscope (Olympus, Japan) at ×40 magnification.

### Mice Nebulized Lung Delivery Infection Model

Wild-type (WT) C57BL/6 female mice (6 weeks old) were purchased from Charles River (Beijing, China). The mice were maintained and bred under Specific pathogen-free (SPF) conditions in the animal facilities of the Academy of Military Medical Sciences. For pulmonary infection, mice were anesthetized and then 50 μl of PBS containing a 1 × 10^8^ CFU dose of GAS was inhaled *via* a nebulized lung delivery. After infection, mice were injected intraperitoneally (i.p.) with 100 μl of luteolin at a dose of 10 mg/kg body weight (Zhang et al., 2021) and luteolin was additionally administered at 24 h intervals for 12 days. The blank control group was not treated with GAS and was separately isolated; the positive control group was given the same volume of PBS according to the same procedure after infection. Each experimental group contained 10 mice, and the survival rate was recorded within 12 days and observed every 24 h.

### Hematoxylin-Eosin Staining of Infected Lung Tissue

For histopathological analysis, mice were sacrificed by cervical dislocation 7 days after infection, each group containing multiple lung tissue samples was fixed in with 4% formaldehyde, stained with hematoxylin and eosin, and observed by OlympusBX53 microscopy.

### Data Analysis

Results are shown as mean ± standard deviation (SD) derived minimally from three independent experiments. A paired two-tailed Student’s *t*-test, one-way or two-way analysis of variance (ANOVA) with Bonferroni multiple comparison test was used to test for significant differences using GraphPad Prism 8.0 software (San Diego, California, United States). Differences were considered statistically significant when *p* < 0.05: *p < 0.05, **p < 0.01, and ***p < 0.001.

## Results

### Luteolin Inhibits the Hemolytic Activity of SLO

**FIGURE 1:**
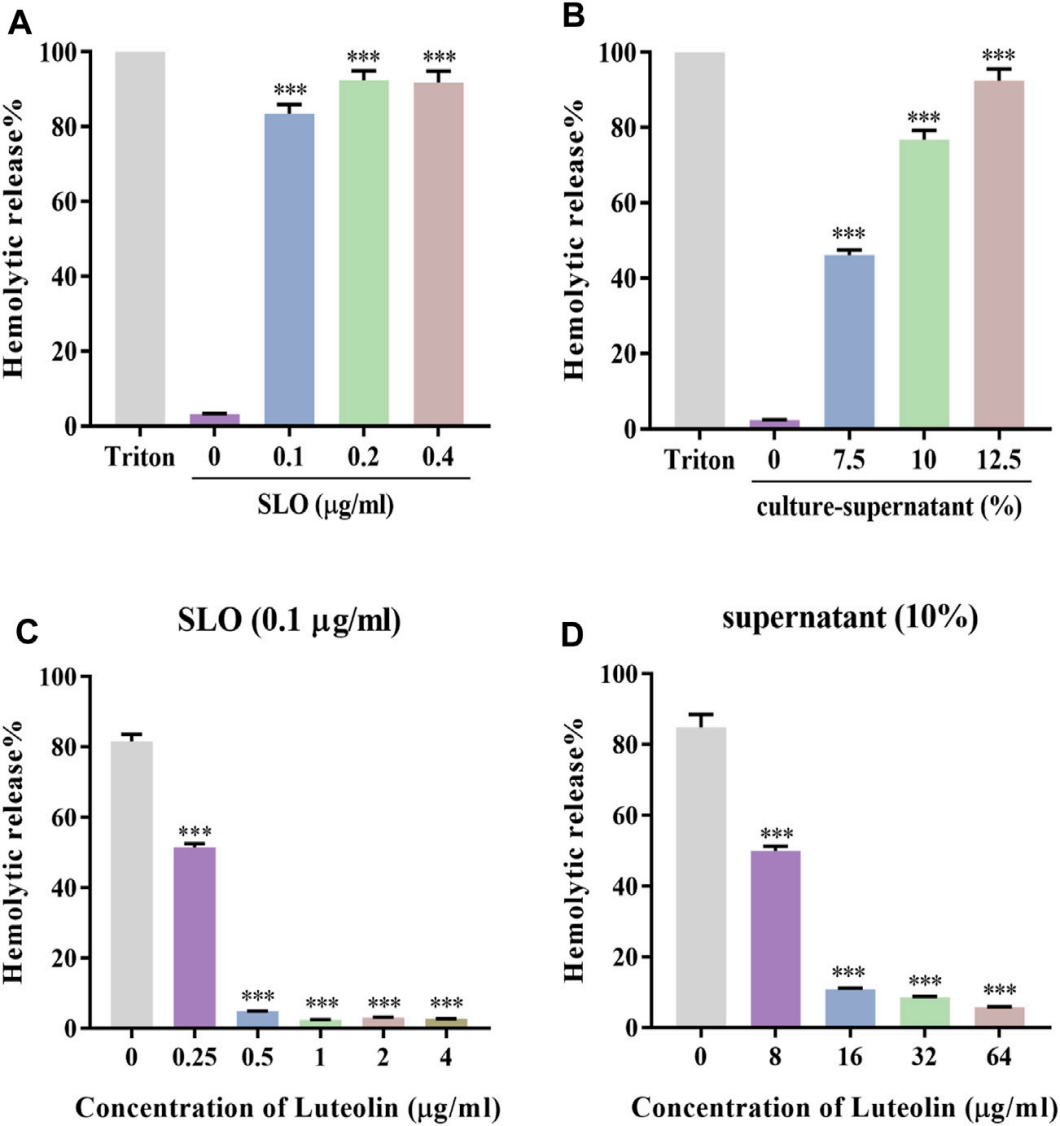
Luteolin affected the hemolytic activity of SLO and culture-supernatant. **(A,B)** RBCs were treated with 0, 0.1, 0.2, and 0.4 μg/ml of SLO or 0%, 7.5%, 10%, and 12.5% of GAS culture-supernatant for 30 min at 37°C. **(C)** Effect of luteolin on RBC lysis induced by SLO. **(D)** Effect of luteolin on the hemolytic activity of GAS culture-supernatant. The OD at 540 nm of each sample was obtained. Results were represented as the percentage of lysed RBCs (assuming as 100% the positive control) derived from three independent experiments ± SD (One-way ANOVA, ***p < 0.001 compared with cells treated with 0.1 μg/ml of SLO in the absence of luteolin).

Luteolin has been shown to have antibacterial activity against several bacterial species. Previous studies have shown that luteolin exhibited anti-GAS activity with an MIC = 128 μg/ml (Siriwong et al., 2015). We found the protective effect of luteolin to be significantly lower than the MIC against SLO-induced cytotoxicity by hemolysis tests. First, a dose-response analysis of the SLO protein (Figure 1A) and GAS culture-supernatant (Figure 1B) hemolytic effects in RBCs was performed. Results showed hemolytic release to be proportional to the concentration of SLO or the percentage of supernatant added, reaching up to 80% hemolysis in RBCs treated with 0.1 μg/ml SLO or 10% (v/v) supernatant, compared to the negative control. SLO or supernatant was incubated with different concentrations of luteolin for 30 min, compared with luteolin-untreated cells exposed to SLO or supernatant, and we found that luteolin likewise inhibited the percentage of hemolytic release in a dose-dependent manner (Figures 1C,D).

### Analysis of the Interactions and Affinity Between Luteolin and SLO

**FIGURE 2:**
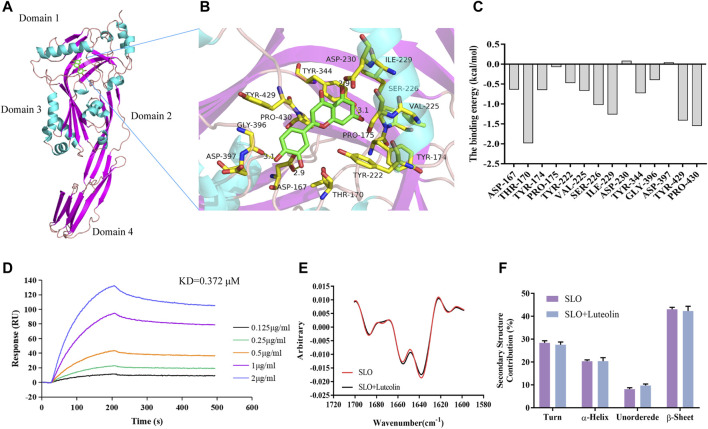
Binding mode of luteolin to SLO. **(A)** Details of the binding site. Amino acid residues in the binding complex (**B)**. **(C)** Energy contributions of the important residues during the binding process. The binding free energy was calculated using the MM-PBSA method. The residues THR-170, TYR-429 and PRO-430 had high binding energy and were critical for the binding of luteolin to SLO. **(D)** SLO was immobilized on an SPR assay chip and luteolin, at the indicated concentrations, was used to determine the binding activity. **(E)** MMS measurement of protein and protein-ligand complex. **(F)** Comparison of the secondary structure composition (%).

Molecular docking technology is to predict the possible binding mode and conformation in the binding site between small molecules (also known as ligands) and protein receptors through the interaction between them, and to estimate the affinity for this specific interaction (Alonso et al., 2006). MD simulations carry out a more intensive conformational search than molecular docking methods do and provide a more accurate representation of protein motions (Morra et al., 2010). Therefore, in order to explore the mechanism of interaction between SLO and luteolin, we employed molecular docking and MD simulation to analyze the SLO—luteolin complex. Luteolin was found to bind to protein domains 1 and 3 of the SLO protein (Figure 2A). The binding site is mainly composed of β pleats, surrounded by helical structures on both sides. It is a semi-open binding site, which has a certain hydrophilicity. The binding energy between luteolin and SLO was −7.2 kcal/mol. The binding force mainly included hydrogen bonding, hydrophobic interactions, and van der Waals forces, and the interacting residues involved mainly included Gly396, Asp167, Asp230, Tyr344, Tyr429, Ile229, Tyr222, and Tyr174 (Figure 2B). The MM-PBSA method was used to calculate the interaction free binding energy to determine the contribution of amino acid residues that promote the binding of luteolin to SLO. The results confirmed a strong interaction between THR-170, TYR-429, PRO-430, and luteolin (Figure 2C), the binding energies of which were −1.9793, −1.544, −1.4026 kcal/mol, respectively.

To verify the molecular interaction between luteolin and streptolysin O, SLO protein was immobilized on a SPR chip and luteolin was used as the mobile phase to study the interaction between them. The results (Figure 2D) showed that luteolin had a high affinity for the SLO protein, with a KD of 0.372 μM (Table 2).

**TABLE 2:** Interacting affinities of luteolin to SLO, SLY, PLY, LLO measured by SPR.

	K_a_ (M^−1^ s^−1^)	K_d_ (s^−1^)	K_D_ (μM)
SLO	1.82e3	6.76e-4	0.372
SLY	2.05e3	1.13e-3	0.554
PLY	1.91e3	1.17e-3	0.613
LLO	1.88e3	8.21e-4	0.437

The 2nd derivative MMS spectra of these samples were overlaid in Figure 2E and the secondary structure composition (%) were compared in Figure 2F. The results clearly show that there was no significant difference in protein structure before and after small molecule binding. Taking the average value of three repeated tests, using the SLO protein as the standard to calculate the similarity, the similarity between the luteolin-SLO complex and SLO protein alone was 97.27%. These results show that luteolin binding did not significantly change the secondary structure of the SLO protein.

### Luteolin Increases the Stability of the SLO Protein and Inhibits Its Polymerization.

**FIGURE 3:**
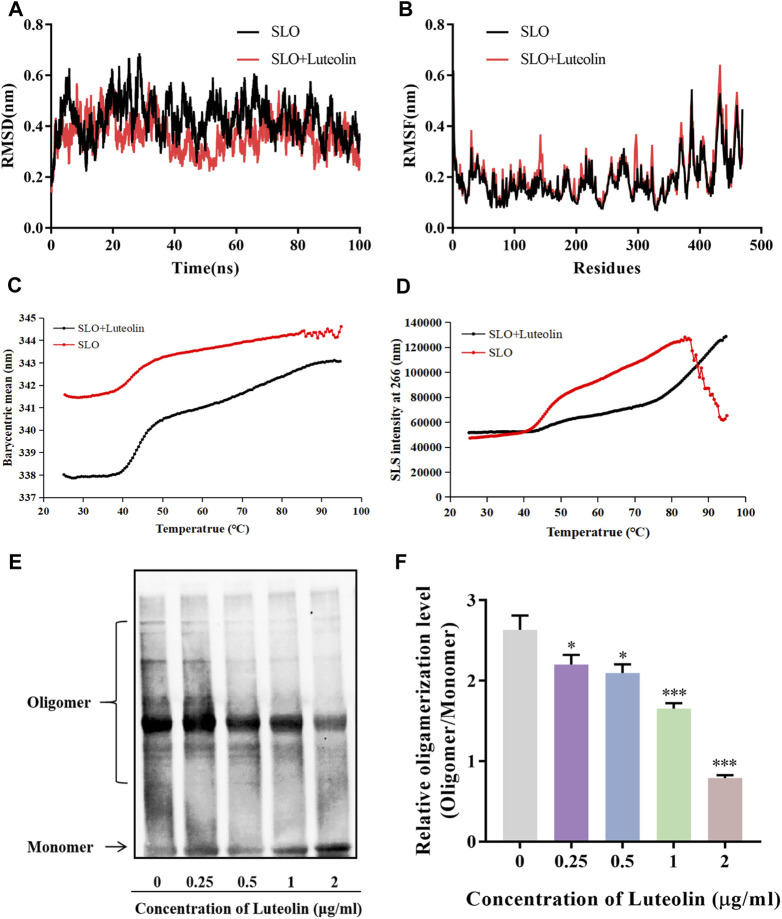
Interactions of SLO with luteolin and effects of luteolin on the protein structure and stability of the SLO protein. **(A,B)** The RMSD and RMSF values of the backbone atoms in the luteolin-SLO complex (red line) and the system containing only the SLO protein (black line) during the MD procedure. **(C,D)** The barycentric mean (BCM) indicates the protein folding state, thermal stability of protein vs. protein-ligand complex, measured by static light scattering (SLS). **(E)** Western blot analysis of luteolin’s effect on the oligomerization of SLO. **(F)** Quantitative densitometric analysis of the oligomerization level (oligomer/monomer) was performed using NIH ImageJ software (GE Healthcare Life Sciences, United Kingdom), with data are presented as mean ± SD (One-way ANOVA) for each group (*n* = 3), *p < 0.05; **p < 0.01; ***p < 0.001 versus positive control.

To further investigate the interaction mechanism between luteolin and SLO, we further analyzed the MD simulation results of SLO protein and luteolin. The root-mean-square deviation (RMSD) curve represents the fluctuation of SLO protein conformation. It can be seen in Figure 3A, that for the small molecule—protein complex containing the ligand (luteolin), the fluctuation of protein conformation during the simulation process was significantly smaller than that of the system containing only protein. These results indicated that binding of luteolin may stabilize the SLO protein’s conformation. Next, we analyzed the root-mean-square-fluctuation (RMSF) (Figure 3B) to explore the conformational changes of side chain residues before and after luteolin binding. We found that there were similar amino acid residue fluctuations between the two groups. And we used the UNcle multi-parameter high-throughput protein stability analysis system to detect changes in SLO protein stability before and after luteolin treatment. The results showed that the melting temperature (Tm) of the SLO protein treated with luteolin (Tm = 43.66°C) increased relative to that of the untreated SLO protein (Tm = 41.25°C) (Figure 3C). The small size and unfolded aggregates of the protein were observed at 266 nm by static light scattering (SLS). The aggregation temperature (Tagg) of the treated SLO protein was 42.49°C, while the of the untreated protein (Tagg) was 38.62°C (Figure 3D). These results suggest that luteolin may help maintain the conformational stability of SLO protein and prevent aggregation. In addition, oligomerization analysis was conducted to determine whether luteolin affects the toxicity of SLO proteins by affecting their pore-forming ability. Early evidence suggests that oligomers of hemolysin proteins loaded into SDS-PAGE are less stable than monomers (Lv et al., 2020). Therefore, the protein concentration (100 μg/ml) used in oligomeric analysis is much higher than that used in the hemolysis assay. The purified SLO protein was mixed with luteolin at different concentrations, and those without added compounds were used as positive controls. As shown in Figures 3E,F, oligomerization decreased in response to high concentrations of luteolin. Compared with hemolysis tests, hemolysis activity was almost completely inhibited by luteolin at the same concentration (2 μg/ml), but the presence of SLO protein oligomers could still be detected, likely due to the higher protein concentration used in this test. We know that the formation of protein oligomers is a coordinated change in conformation of the protein domain from the α-helices to the insertion of two β-sheets into the membrane to form a β-barrel structured pore in the membrane (Heuck et al., 2003). Therefore, we speculate that luteolin might play a role in inhibiting SLO toxicity by affecting stability of the protein structure, thus inhibiting the SLO protein oligomerization.

### Luteolin Binds to Other CDC Family Proteins and Inhibits Their Hemolytic Activity

**FIGURE 4:**
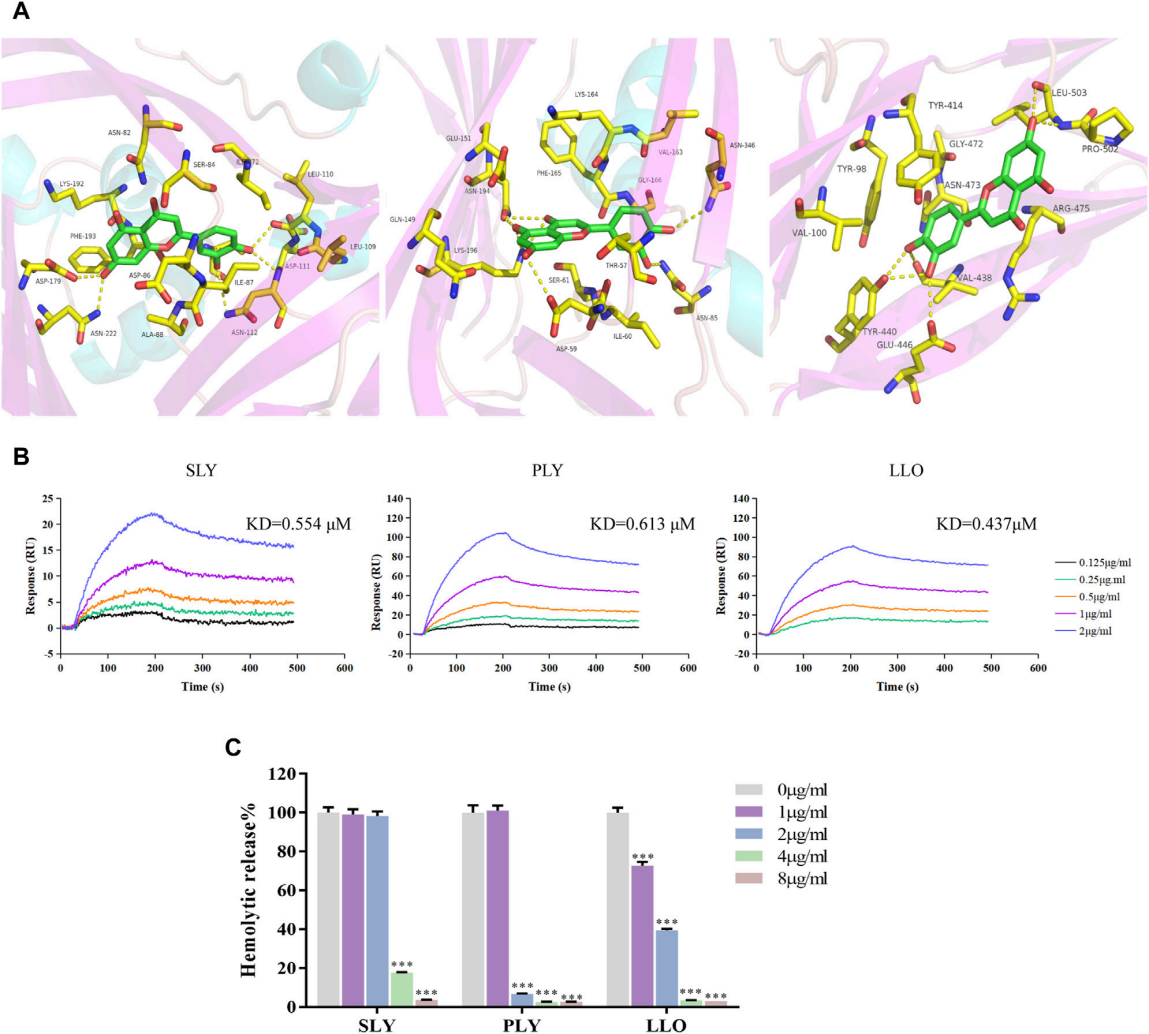
Luteolin inhibits the hemolytic activity of SLY, PLY and LLO proteins. **(A)** The binding mode of luteolin with SLY, PLY and LLO. **(B)** Luteolin combined with SLY, PLY, and LLO. **(C)** Luteolin affects the hemolytic activity of SLY, PLY and LLO proteins. *p < 0.05; **p < 0.01; ***p < 0.001; ns. indicates results were not significant.

Molecular docking results showed the binding mode of luteolin with SLY, PLY, and LLO proteins (Figure 4A). We compared the important residues resulting from the combination of SLO and luteolin with the amino acid sequences of other CDCs. Interestingly, we found no conserved residues between them. But the SPR results (Figure 4B) showed that luteolin also had high affinities for SLY, PLY, and LLO proteins, with affinity constants of 0.554, 0.613, and 0.437 μM (Table 2), respectively. Additionally, our results indicated that luteolin also inhibited the hemolytic activity of these proteins (Figure 4C), and luteolin concentrations that completely inhibited the hemolytic activities of SLY, PLY, and LLO proteins were 8, 4, and 4 μg/ml, respectively.

### Luteolin Protects HEp-2 Epithelial Cells From SLO-Induced Cytotoxicity

**FIGURE 5:**
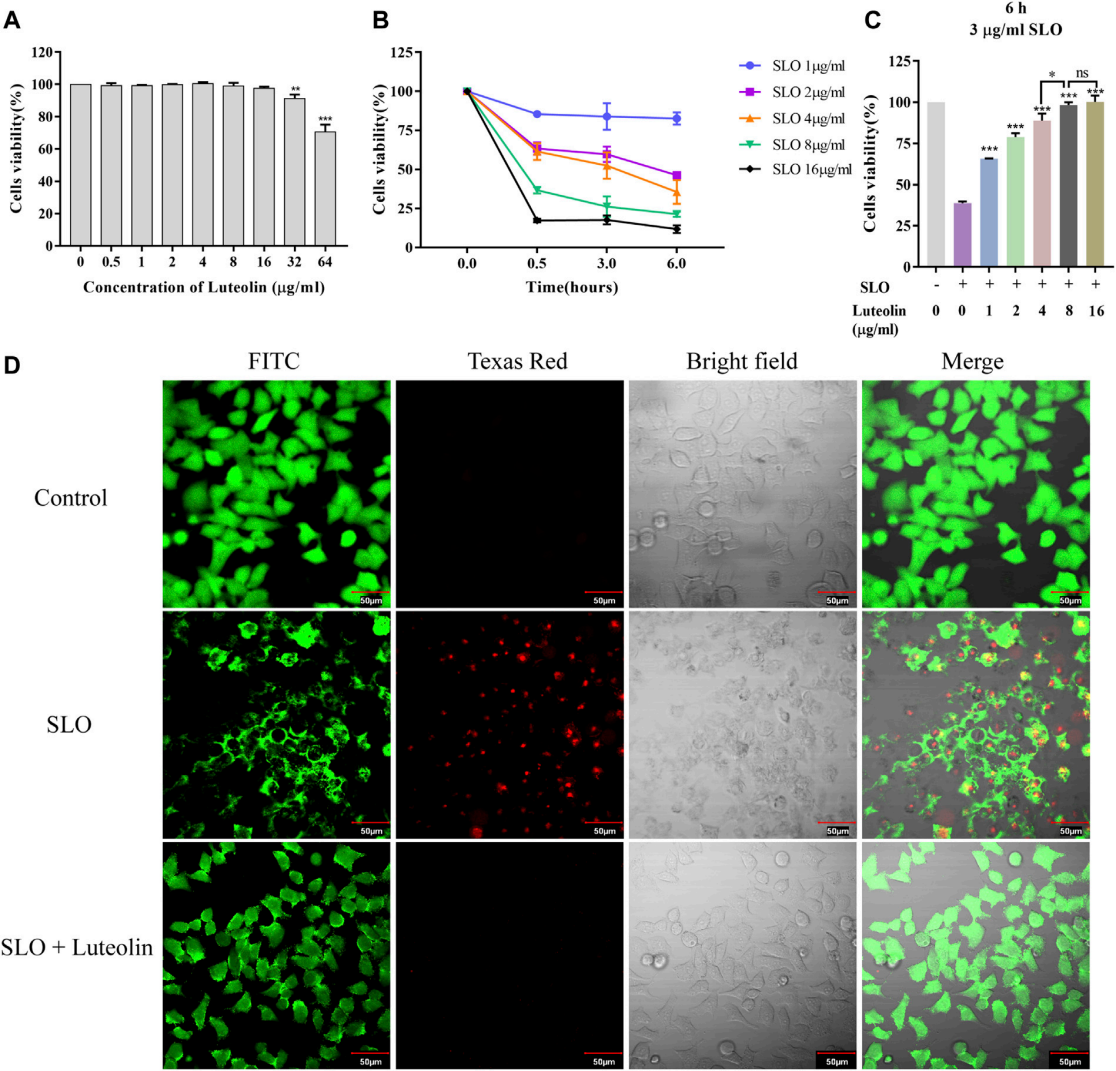
Effect of Luteolin on SLO toxin-mediated pathology in human HEp-2 cells. **(A)** Cytotoxicity of luteolin for 24 h. **(B)** Dose-response and time-course experiments to evaluate SLO cytotoxic effects in HEp-2 cells. Cells were treated with SLO at concentrations of 1, 2, 4, 8, and 16 μg/ml for 0, 0.5, 3, and 6 h, respectively. Cell viability was determined by the CCK-8 assay, and the cell viability of the untreated group was defined as 100%. Data represent the mean value ± SD derived from three replicates. **(C)** HEp-2 cells were treated for 6 h with 3 μg/ml of SLO, in the absence or presence of luteolin. The cell viability was determined by the CCK-8 assay, and the cell viability of the untreated group was defined as 100%. Data represent the mean value ± SD derived from three replicates (Two-way ANOVA and Tukey’s post test). *p < 0.05; **p < 0.01; ***p < 0.001, with ns. used to indicate results were not significant. **(D)** HEp-2 cells were treated with 3 μg/ml of SLO, in the absence or presence of luteolin (8 μg/ml), for 6 h. Cells were stained with FITC fluorescent cell dye solution (green signal) to visualize living cells. Texas Red staining, which mainly produces nuclear red fluorescence in cells with damaged cell membranes, was used to visualize damaged cells.

In order to neutralize the toxicity of SLO without inducing cytotoxicity, preliminary experiments were carried out on HEp-2 cells with different concentrations of luteolin to determine the optimal concentration to use. The results showed that the effective concentration of luteolin lower than 16 μg/ml could be used for toxicity test intervention without affecting cell viability (Figure 5A).

Next, dose-response and time-course analyses of SLO cytotoxicity in HEp-2 cells grown in FBS free medium was performed. Cells treatment with SLO caused a dose- and time-dependent decrease in cell survival (Figure 5B). On the basis of the IC50 values (3 μg/ml) calculated, we treated HEp-2 cells with 3 μg/ml of SLO for 6 h. HEp-2 cells exposed to 3 μg/ml SLO and co-incubated with tolerated doses of luteolin for 6 h showed increased cell viability compared with luteolin-untreated cells (Figure 5C), the results showed that the cell viability was increased by 26.9%, 40.0%, 50.0%, 59.4%, and 61.4%, respectively. Moreover, we compared the concentrations of luteolin and found that luteolin of 8 μg/ml was the lowest effective concentration to still completely resist the toxicity of SLO protein. In addition, we investigated whether luteolin could protect HEp-2 cells from SLO dependent permeability. Intact living cells were visualized by staining with FITC fluorescent cell dye solution (green signal); dead cells were visualized using Texas Red stain, which mainly produces nuclear red fluorescence in cells with damaged cell membranes, but is not taken up by living cells. The results showed that the SLO protein induced cell permeability, and the presence of luteolin in the culture medium inhibited SLO induced permeability (Figure 5D).

### Luteolin Provides Protection Against GAS Pneumonia in Mice

**FIGURE 6:**
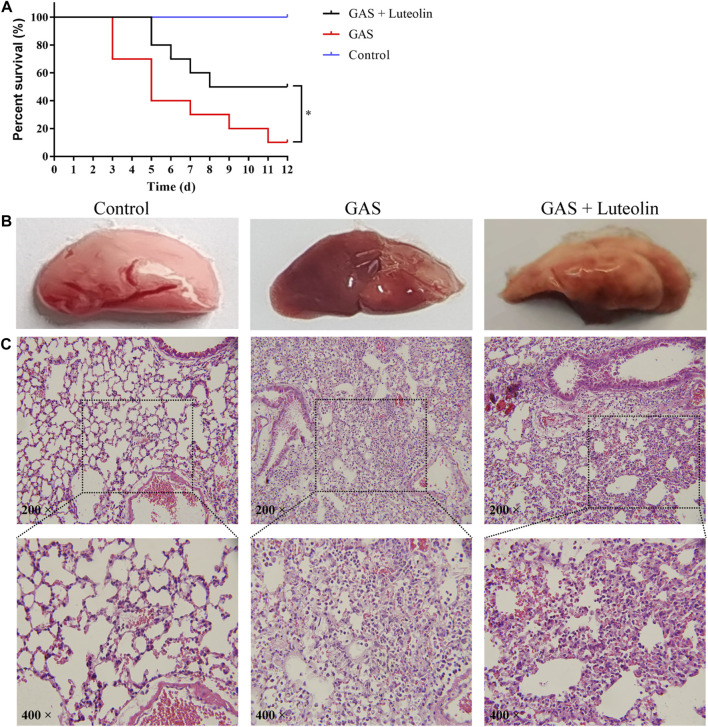
Luteolin protects mice against GAS pneumonia. **(A)** The influence of luteolin on the mortality of GAS-infected mice. GAS, GAS + luteolin, and Control groups of mice inhaled 1 × 10^8^ CFU dose of GAS *via* a nebulized lung delivery, and the mortality of mice was supervised for 12 days. Data are representative of three independent experiments (*n* = 10 mice in the per group) and are shown as mean ± SD (Kaplan-Meier tests). *p < 0.05 versus control. **(B,C)** Pathologic and histopathological changes in lung tissue. Lung tissue from GAS-infected mice (7 days after infection). Lung tissue was stained with H&E (original magnification ×200).

Based on the above-presented experimental conclusions, we further verified whether luteolin has a similar protective effect in mice infected with GAS. First, mortality due to pneumonia caused by GAS was monitored every 24 h after infection over a 12-day time course. The survival curve is shown in Figure 6A. Compared with the healthy control group, the survival rate of mice infected with GAS was 10%. However, the survival rate increased to 50% when infected mice were treated with luteolin. Pathological analysis of lung tissue was performed on each group of mice to evaluate the mitigating effect of luteolin on lung injury. Pathological examination revealed that lung tissue from infected mice was dark red and showed severe congestion and edema. In contrast, mice treated with luteolin showed a light pink lesion with focal infection (Figure 6B). Pathological assessment of lung tissue sections showed severe destruction of lung tissue and inflammatory cell infiltration in infected mice. After luteolin treatment, lung tissue damage was reduced, and the inflammatory response was relieved (Figure 6C). The results of this experiment indicate that luteolin treatment can improve the pulmonary inflammatory injury induced by GAS, delay and decrease the aggravation of the disease.

## Discussion

At present, drug resistance of pathogenic bacteria is becoming increasingly widespread due to the extensive, massive, long-term, and non-standard use of antibiotics, producing one of the largest medical challenges of the 21st century (Group et al., 2012; Shulman et al., 2012; Hedin et al., 2014; Yu et al., 2020). As a result, the failure rate for treating related infectious diseases is increasing (Cohen, 2004). Several new antimicrobial strategies against not only GAS but other bacteria, including interfering with bacterial toxicity and/or intercellular signaling pathways, have been identified through further research and understanding (Rasko and Sperandio, 2010). For example, human serum albumin can bind C. *difficile* TcdA and TcdB toxins, reduced bacterial toxin dependent infection, and helped protect the host cell (Di Bella et al., 2015; di Masi et al., 2018). Honokiol inhibited the secretion and hemolytic activity of Hla, which reduced the Hla-induced inflammatory response and the damaging effects of *Staphylococcus aureus* infection on cells (Guo et al., 2019). Quercetin significantly reduced PLY-induced hemolytic activity, cytotoxicity, and cell damage by suppressing the formation of oligomers and alleviated the pathological damage to lung tissue and the release of cytokines in mice infected with *Streptococcus pneumoniae* (Lv et al., 2020). These compounds have different modes of action than traditional bactericidal or bacteriostatic chemicals, which expands the arsenal of antibacterial treatments to a certain extent.

In this study, we discussed the detoxification mechanism of the small plant-based ligand luteolin against the SLO protein, one of the key virulence factors of GAS. SLO induces eukaryotic cell lysis, resists neutrophil phagocytosis and clearance of GAS, inhibits neutrophil oxidative burst, and blocks neutrophil degranulation, interleukin-8 secretion, and reactivity, thereby contributing to the pathogenesis of streptococcal infection (Uchiyama et al., 2015), and thus has increasingly becoming a promising potential drug target for the treatment of streptococcal infection (Arzanlou and Bohlooli, 2010; Turner et al., 2020).

Some studies have demonstrated that luteolin has increased antibacterial effects relative to many commonly used compounds, and can have a synergistic effect when combined with ceftazidime (Siriwong et al., 2015). Additionally, it also has obvious bacteriostatic effects on *Bacillus subtilis*, *Staphylococcus aureus*, *Pseudomonas fluorescens*, *Escherichia coli*, *Enterobacter cloacae*, and *Trueperella pyogenes* (Lv et al., 2009; Guo et al., 2020; Geng et al., 2021; Qian et al., 2021). At present, luteolin has been used in food packaging films to exert long-term antioxidant and antibacterial activities during food storage (Bi et al., 2021). Scientists are constantly trying various methods to increase the bioavailability of luteolin, one example is the use of luteolin-loaded Methoxy poly(ethylene glycol)-poly(lactide) micelles that can significantly promote bacterial clearance while reducing inflammatory infiltration in a mouse model of pulmonary infection caused by *Klebsiella pneumoniae* (Miao et al., 2021). Recent studies have demonstrated that luteolin can alleviate olfactory dysfunction after COVID-19, which provides further support for the clinical application of luteolin (D'Ascanio et al., 2021). In addition, through bioinformatics analysis such as TCGA-RNA-seq and network pharmacology, the researchers concluded that luteolin may play a clinical role in anti-PC and 2019-nCoV disease by regulating the activities of core genes (MPO and FOS) (Ye et al., 2021).

Here, we demonstrated that luteolin binds the SLO protein at domain 1 and 3 with high affinity. These domains consists of several α-helices and a number of loops surrounding a core β-sheet (Feil et al., 2014), and conformational changes are key to the formation of protein polymers and pores (Hotze et al., 2012). The results of this study further demonstrate that luteolin strengthened the conformational stability of the SLO protein and inhibited the formation of its polymers, thus weakening its pore-forming activity. We also carried out point mutations on residues involved in SLO binding to luteolin, purified the protein, and then verified the hemolytic functional experiment. At the same time, we carried out experiments on the interaction between luteolin and the point mutant protein, but we did not find obvious changes in protein function and intermolecular interaction (data not shown). Therefore, we speculate that the mutation of a single residue may not play a key role and may require the joint action of multiple residues. Meanwhile, luteolin inhibited the hemolysis activity of the SLO protein in a concentration-dependent manner. Luteolin also played a protective role by preventing SLO induced permeability of epithelial cell membranes.

Meanwhile, our experiments verified that luteolin also interacted with other CDC family proteins, including SLY, PLY, and LLO, and inhibited their hemolytic activity. Although the binding residues among them are not very consistent through further analysis by molecular docking, luteolin has high affinities for SLY, PLY, and LLO proteins. Previous studies had shown that luteolin can play a targeted role against *L. monocytogenes* mediated infection by inhibiting translation of the LLO protein and reducing its expression, however, luteolin was not found to affect the release of PLY (Wang et al., 2019). However, our study demonstrated that there are certain interactions between luteolin and CDC family proteins that can inhibit some of their functional activities, thus enriching the antibacterial targets of luteolin. Moreover, the results of *in vivo* experiments concluded that luteolin intervention could improve the inflammatory damage of mice with pulmonary infection induced by GAS and improve the survival rate. This may also provide a preliminary scientific basis for the treatment of clinically relevant infectious diseases.

Studies on *in vivo* infection showed that cytotoxicity of the SLO protein could protect GAS from phagocytic killing by host cells and enhance its virulence, which was significantly related to the pathogenicity of GAS and severity of disease (Sierig et al., 2003; Zhu et al., 2017). Our results show that luteolin can significantly inhibit the pathogenicity of GAS, improve damage associated with inflammation. These results may be related to the interaction between luteolin and SLO protein. In conclusion, luteolin may be a potential SLO-targeting, indirectly antibacterial compound, which reduces the selection pressure against bacteria and the probability of drug resistant bacteria evolving. The very promising medicinal value of luteolin lays a foundation for further study of its anti-virulence mechanisms.

## Data Availability

The original contributions presented in the study are included in the article, further inquiries can be directed to the corresponding authors.

## References

[B1] Alonso H. Bliznyuk A. A. Gready J. E. (2006). Combining Docking and Molecular Dynamic Simulations in Drug Design. Med. Res. Rev. 26 (5), 531–568. 10.1002/med.20067 16758486

[B2] Arzanlou M. Bohlooli S. (2010). Inhibition of Streptolysin O by Allicin - an Active Component of Garlic. J. Med. Microbiol. 59 (Pt 9), 1044–1049. 10.1099/jmm.0.019539-0 20538890

[B3] Bi F. Qin Y. Chen D. Kan J. Liu J. (2021). Development of Active Packaging Films Based on Chitosan and Nano-Encapsulated Luteolin. Int. J. Biol. Macromol. 182, 545–553. 10.1016/j.ijbiomac.2021.04.063 33857507

[B4] Biharee A. Sharma A. Kumar A. Jaitak V. (2020). Antimicrobial Flavonoids as a Potential Substitute for Overcoming Antimicrobial Resistance. Fitoterapia 146, 104720. 10.1016/j.fitote.2020.104720 32910994

[B5] Bricker A. L. Cywes C. Ashbaugh C. D. Wessels M. R. (2002). NAD+-glycohydrolase Acts as an Intracellular Toxin to Enhance the Extracellular Survival of Group A Streptococci. Mol. Microbiol. 44 (1), 257–269. 10.1046/j.1365-2958.2002.02876.x 11967084

[B6] Chen D. Bi A. Dong X. Jiang Y. Rui B. Liu J. (2014). Luteolin Exhibits Anti-inflammatory Effects by Blocking the Activity of Heat Shock Protein 90 in Macrophages. Biochem. Biophys. Res. Commun. 443 (1), 326–332. 10.1016/j.bbrc.2013.11.122 24321097

[B7] Clarke J. Baltazar M. Alsahag M. Panagiotou S. Pouget M. Paxton W. A. (2021). Streptolysin O Concentration and Activity Is Central to *In Vivo* Phenotype and Disease Outcome in Group A Streptococcus Infection. Sci. Rep. 11 (1), 19011. 10.1038/s41598-021-97866-4 34561464 PMC8463576

[B8] Cohen R. (2004). Defining the Optimum Treatment Regimen for Azithromycin in Acute Tonsillopharyngitis. Pediatr. Infect. Dis. J. 23 (2 Suppl. l), S129–S134. 10.1097/01.inf.0000112527.33870.0d 14770076

[B9] D'Ascanio L. Vitelli F. Cingolani C. Maranzano M. Brenner M. Di Stadio A. J. E. (2021). Randomized Clinical Trial "olfactory Dysfunction after COVID-19: Olfactory Rehabilitation Therapy vs. Intervention Treatment with Palmitoylethanolamide and Luteolin. Prelim. results 25 (11), 4156–4162. 10.26355/eurrev_202106_26059 34156697

[B10] Dal Peraro M. van der Goot F. G. (2016). Pore-forming Toxins: Ancient, but Never Really Out of Fashion. Nat. Rev. Microbiol. 14 (2), 77–92. 10.1038/nrmicro.2015.3 26639780

[B11] Di Bella S. di Masi A. Turla S. Ascenzi P. Gouliouris T. Petrosillo N. (2015). The Protective Role of Albumin in *Clostridium difficile* Infection: A Step toward Solving the Puzzle. Infect. Control Hosp. Epidemiol. 36 (12), 1478–1479. 10.1017/ice.2015.221 26456662

[B12] di Masi A. Leboffe L. Polticelli F. Tonon F. Zennaro C. Caterino M. (2018). Human Serum Albumin Is an Essential Component of the Host Defense Mechanism against *Clostridium difficile* Intoxication. J. Infect. Dis. 218 (9), 1424–1435. 10.1093/infdis/jiy338 29868851

[B13] Dong J. Liu Y. Xu N. Yang Q. Ai X. (2018). Morin Protects Channel Catfish from Aeromonas Hydrophila Infection by Blocking Aerolysin Activity. Front. Microbiol. 9, 2828. 10.3389/fmicb.2018.02828 30519232 PMC6258893

[B14] Dong J. Zhang L. Liu Y. Xu N. Zhou S. Yang Y. (2021). Luteolin Decreases the Pathogenicity of Aeromonas Hydrophila via Inhibiting the Activity of Aerolysin. Virulence 12 (1), 165–176. 10.1080/21505594.2020.1867455 33372840 PMC7781616

[B15] Feil S. C. Ascher D. B. Kuiper M. J. Tweten R. K. Parker M. W. (2014). Structural Studies of Streptococcus Pyogenes Streptolysin O Provide Insights into the Early Steps of Membrane Penetration. J. Mol. Biol. 426 (4), 785–792. 10.1016/j.jmb.2013.11.020 24316049 PMC4323271

[B16] Geng Y. F. Yang C. Zhang Y. Tao S. N. Mei J. Zhang X. C. (2021). An Innovative Role for Luteolin as a Natural Quorum Sensing Inhibitor in *Pseudomonas aeruginosa* . Life Sci. 274, 119325. 10.1016/j.lfs.2021.119325 33713665

[B17] Group E. S. T. G. Pelucchi C. Grigoryan L. Galeone C. Esposito S. Huovinen P. (2012). Guideline for the Management of Acute Sore Throat. Clin. Microbiol. Infect. 18 Suppl 1 (Suppl. 1), 1–28. 10.1111/j.1469-0691.2012.03766.x 22432746

[B18] Guo N. Liu Z. Yan Z. Liu Z. Hao K. Liu C. (2019). Subinhibitory Concentrations of Honokiol Reduce α-Hemolysin (Hla) Secretion by *Staphylococcus aureus* and the Hla-Induced Inflammatory Response by Inactivating the NLRP3 Inflammasome. Emerg. Microbes Infect. 8 (1), 707–716. 10.1080/22221751.2019.1617643 31119985 PMC6534259

[B19] Guo Y. Liu Y. Zhang Z. Chen M. Zhang D. Tian C. (2020). The Antibacterial Activity and Mechanism of Action of Luteolin against Trueperella Pyogenes. Infect. Drug Resist 13, 1697–1711. 10.2147/IDR.S253363 32606820 PMC7293968

[B20] Hedin K. Strandberg E. L. Gröndal H. Brorsson A. Thulesius H. André M. (2014). Management of Patients with Sore Throats in Relation to Guidelines: an Interview Study in Sweden. Scand. J. Prim. Health Care 32 (4), 193–199. 10.3109/02813432.2014.972046 25363143 PMC4278394

[B21] Heuck A. P. Tweten R. K. Johnson A. E. (2003). Assembly and Topography of the Prepore Complex in Cholesterol-dependent Cytolysins. J. Biol. Chem. 278 (33), 31218–31225. 10.1074/jbc.M303151200 12777381

[B22] Hotze E. M. Wilson-Kubalek E. Farrand A. J. Bentsen L. Parker M. W. Johnson A. E. (2012). Monomer-Monomer Interactions Propagate Structural Transitions Necessary for Pore Formation by the Cholesterol-dependent Cytolysins. J. Biol. Chem. 287 (29), 24534–24543. 10.1074/jbc.M112.380139 22645132 PMC3397878

[B23] Hu R. Barbault F. Maurel F. Delamar M. Zhang R. (2010). Molecular Dynamics Simulations of 2-Amino-6-Arylsulphonylbenzonitriles Analogues as HIV Inhibitors: Interaction Modes and Binding Free Energies. Chem. Biol. Drug Des. 76 (6), 518–526. 10.1111/j.1747-0285.2010.01028.x 20942836

[B24] Jiang L. Yi T. Shen Z. Teng Z. Wang J. (2019). Aloe-emodin Attenuates *Staphylococcus aureus* Pathogenicity by Interfering with the Oligomerization of α-Toxin. Front. Cell. Infect. Microbiol. 9, 157. 10.3389/fcimb.2019.00157 31157174 PMC6530610

[B25] Jogalekar A. S. Reiling S. Vaz R. J. (2010). Identification of Optimum Computational Protocols for Modeling the Aryl Hydrocarbon Receptor (AHR) and its Interaction with Ligands. Bioorg Med. Chem. Lett. 20 (22), 6616–6619. 10.1016/j.bmcl.2010.09.019 20875740

[B26] Lin Y. Shi R. Wang X. Shen H. M. (2008). Luteolin, a Flavonoid with Potential for Cancer Prevention and Therapy. Curr. Cancer Drug Targets 8 (7), 634–646. 10.2174/156800908786241050 18991571 PMC2615542

[B27] López-Lázaro M. (2009). Distribution and Biological Activities of the Flavonoid Luteolin. Mini Rev. Med. Chem. 9 (1), 31–59. 10.2174/138955709787001712 19149659

[B28] Los F. C. Randis T. M. Aroian R. V. Ratner A. J. (2013). Role of Pore-Forming Toxins in Bacterial Infectious Diseases. Microbiol. Mol. Biol. Rev. 77 (2), 173–207. 10.1128/MMBR.00052-12 23699254 PMC3668673

[B29] Lu G. Xu L. Zhang P. Dou X. Yu J. Feng H. (2019). Betulin Efficiently Suppresses the Process of an Experimental Listeria Monocytogenes Infection as an Antagonist against Listeriolysin O. Fitoterapia 139, 104409. 10.1016/j.fitote.2019.104409 31698059

[B30] Lukoyanova N. Hoogenboom B. W. Saibil H. R. (2016). The Membrane Attack Complex, Perforin and Cholesterol-dependent Cytolysin Superfamily of Pore-Forming Proteins. J. Cell. Sci. 129 (11), 2125–2133. 10.1242/jcs.182741 27179071

[B31] Lv P. C. Li H. Q. Xue J. Y. Shi L. Zhu H. L. (2009). Synthesis and Biological Evaluation of Novel Luteolin Derivatives as Antibacterial Agents. Eur. J. Med. Chem. 44 (2), 908–914. 10.1016/j.ejmech.2008.01.013 18313801

[B32] Lv Q. Zhang P. Quan P. Cui M. Liu T. Yin Y. (2020). Quercetin, a Pneumolysin Inhibitor, Protects Mice against Streptococcus Pneumoniae Infection. Microb. Pathog. 140, 103934. 10.1016/j.micpath.2019.103934 31862394

[B33] Martin J. M. Green M. (2006). Group A streptococcus. Semin. Pediatr. Infect. Dis. 17 (3), 140–148. 10.1053/j.spid.2006.07.001 16934708

[B34] Miao J. Lin F. Huang N. Teng Y. (2021). Improving Anti-inflammatory Effect of Luteolin with Nano-Micelles in the Bacteria-Induced Lung Infection. J. Biomed. Nanotechnol. 17 (6), 1229–1241. 10.1166/jbn.2021.3101 34167635

[B35] Mitchell T. J. (2003). The Pathogenesis of Streptococcal Infections: from Tooth Decay to Meningitis. Nat. Rev. Microbiol. 1 (3), 219–230. 10.1038/nrmicro771 15035026

[B36] Morra G. Genoni A. Neves M. A. Merz K. M. Colombo G. (2010). Molecular Recognition and Drug-Lead Identification: What Can Molecular Simulations Tell Us? Curr. Med. Chem. 17 (1), 25–41. 10.2174/092986710789957797 19941480

[B37] Morris G. M. Huey R. Lindstrom W. Sanner M. F. Belew R. K. Goodsell D. S. (2009). AutoDock4 and AutoDockTools4: Automated Docking with Selective Receptor Flexibility. J. Comput. Chem. 30 (16), 2785–2791. 10.1002/jcc.21256 19399780 PMC2760638

[B38] Mozola C. C. Caparon M. G. (2015). Dual Modes of Membrane Binding Direct Pore Formation by Streptolysin O. Mol. Microbiol. 97 (6), 1036–1050. 10.1111/mmi.13085 26059530 PMC4692278

[B39] Nelson G. E. Pondo T. Toews K. A. Farley M. M. Lindegren M. L. Lynfield R. (2016). Epidemiology of Invasive Group A Streptococcal Infections in the United States, 2005-2012. Clin. Infect. Dis. 63 (4), 478–486. 10.1093/cid/ciw248 27105747 PMC5776658

[B40] Qian W. Fu Y. Liu M. Zhang J. Wang W. Li J. (2021). Mechanisms of Action of Luteolin against Single- and Dual-Species of *Escherichia coli* and *Enterobacter cloacae* and its Antibiofilm Activities. Appl. Biochem. Biotechnol. 193 (5), 1397–1414. 10.1007/s12010-020-03330-w 33009585

[B41] Rasko D. A. Sperandio V. (2010). Anti-virulence Strategies to Combat Bacteria-Mediated Disease. Nat. Rev. Drug Discov. 9 (2), 117–128. 10.1038/nrd3013 20081869

[B42] Rossjohn J. Polekhina G. Feil S. C. Morton C. J. Tweten R. K. Parker M. W. (2007). Structures of Perfringolysin O Suggest a Pathway for Activation of Cholesterol-dependent Cytolysins. J. Mol. Biol. 367 (5), 1227–1236. 10.1016/j.jmb.2007.01.042 17328912 PMC3674820

[B43] Rungrotmongkol T. Nunthaboot N. Malaisree M. Kaiyawet N. Yotmanee P. Meeprasert A. (2010). Molecular Insight into the Specific Binding of ADP-Ribose to the nsP3 Macro Domains of Chikungunya and Venezuelan Equine Encephalitis Viruses: Molecular Dynamics Simulations and Free Energy Calculations. J. Mol. Graph Model 29 (3), 347–353. 10.1016/j.jmgm.2010.09.010 21036084

[B44] Shewell L. K. Harvey R. M. Higgins M. A. Day C. J. Hartley-Tassell L. E. Chen A. Y. (2014). The Cholesterol-dependent Cytolysins Pneumolysin and Streptolysin O Require Binding to Red Blood Cell Glycans for Hemolytic Activity. Proc. Natl. Acad. Sci. U. S. A. 111 (49), E5312–E5320. 10.1073/pnas.1412703111 25422425 PMC4267402

[B45] Shulman S. T. Bisno A. L. Clegg H. W. Gerber M. A. Kaplan E. L. Lee G. (2012). Clinical Practice Guideline for the Diagnosis and Management of Group A Streptococcal Pharyngitis: 2012 Update by the Infectious Diseases Society of America. Clin. Infect. Dis. 55 (10), e86–102. 10.1093/cid/cis629 22965026 PMC7108032

[B46] Sierig G. Cywes C. Wessels M. R. Ashbaugh C. D. (2003). Cytotoxic Effects of Streptolysin O and Streptolysin S Enhance the Virulence of Poorly Encapsulated Group a Streptococci. Infect. Immun. 71 (1), 446–455. 10.1128/IAI.71.1.446-455.2003 12496195 PMC143243

[B47] Siriwong S. Thumanu K. Hengpratom T. Eumkeb G. (2015). Synergy and Mode of Action of Ceftazidime Plus Quercetin or Luteolin on Streptococcus Pyogenes. Evid. Based Complement. Altern. Med. 2015, 759459. 10.1155/2015/759459 PMC4631891 26576195

[B48] Steer A. C. Danchin M. H. Carapetis J. R. (2007). Group A Streptococcal Infections in Children. J. Paediatr. Child. Health 43 (4), 203–213. 10.1111/j.1440-1754.2007.01051.x 17444820

[B49] Turner M. L. Owens S. E. Sheldon I. M. (2020). Glutamine Supports the Protection of Tissue Cells against the Damage Caused by Cholesterol-dependent Cytolysins from Pathogenic Bacteria. PLoS One 15 (3), e0219275. 10.1371/journal.pone.0219275 32163417 PMC7067430

[B50] Tweten R. K. (2005). Cholesterol-dependent Cytolysins, a Family of Versatile Pore-Forming Toxins. Infect. Immun. 73 (10), 6199–6209. 10.1128/IAI.73.10.6199-6209.2005 16177291 PMC1230961

[B51] Uchiyama S. Döhrmann S. Timmer A. M. Dixit N. Ghochani M. Bhandari T. (2015). Streptolysin O Rapidly Impairs Neutrophil Oxidative Burst and Antibacterial Responses to Group A Streptococcus. Front. Immunol. 6, 581. 10.3389/fimmu.2015.00581 26635795 PMC4644796

[B52] van Pee K. Neuhaus A. D'Imprima E. Mills D. J. Kühlbrandt W. Yildiz Ö. (2017). CryoEM Structures of Membrane Pore and Prepore Complex Reveal Cytolytic Mechanism of Pneumolysin. Elife 6. 10.7554/eLife.23644 PMC5437283 28323617

[B53] Vita G. M. De Simone G. Leboffe L. Montagnani F. Mariotti D. Di Bella S. (2020). Human Serum Albumin Binds Streptolysin O (SLO) Toxin Produced by Group A Streptococcus and Inhibits its Cytotoxic and Hemolytic Effects. Front. Immunol. 11, 507092. 10.3389/fimmu.2020.507092 33363530 PMC7752801

[B54] Vorontsov II Miyashita O. (2011). Crystal Molecular Dynamics Simulations to Speed up MM/PB(GB)SA Evaluation of Binding Free Energies of Di-mannose Deoxy Analogs with P51G-M4-Cyanovirin-N. J. Comput. Chem. 32 (6), 1043–1053. 10.1002/jcc.21683 20949512

[B55] Walker M. J. Barnett T. C. McArthur J. D. Cole J. N. Gillen C. M. Henningham A. (2014). Disease Manifestations and Pathogenic Mechanisms of Group A Streptococcus. Clin. Microbiol. Rev. 27 (2), 264–301. 10.1128/CMR.00101-13 24696436 PMC3993104

[B56] Wang J. Liu S. Liu B. Niu X. Deng X. (2019). Luteolin Inhibits Listeriolysin O Translation by Directly Targeting the Coding Region of the Hly mRNA. Front. Microbiol. 10, 1496. 10.3389/fmicb.2019.01496 31312194 PMC6614183

[B57] Ye Y. Huang Z. Chen M. Mo Y. Mo Z. (2021). Luteolin Potentially Treating Prostate Cancer and COVID-19 Analyzed by the Bioinformatics Approach: Clinical Findings and Drug Targets. Front. Endocrinol. (Lausanne) 12, 802447. 10.3389/fendo.2021.802447 35178029 PMC8844187

[B58] Yu D. Zheng Y. Yang Y. (2020). Is There Emergence of β-Lactam Antibiotic-Resistant Streptococcus Pyogenes in China? Infect. Drug Resist 13, 2323–2327. 10.2147/IDR.S261975 32765008 PMC7369151

[B59] Zhang B. C. Zhang C. W. Wang C. Pan D. F. Xu T. D. Li D. Y. (2016). Luteolin Attenuates Foam Cell Formation and Apoptosis in Ox-LDL-Stimulated Macrophages by Enhancing Autophagy. Cell. Physiol. Biochem. 39 (5), 2065–2076. 10.1159/000447902 27825167

[B60] Zhang D. Gao X. Song X. Zhou W. Hong W. Tian C. (2019). Luteolin Showed a Resistance Elimination Effect on Gentamicin by Decreasing MATE mRNA Expression in Trueperella Pyogenes. Microb. Drug Resist 25 (4), 619–626. 10.1089/mdr.2018.0097 30431396

[B61] Zhang Y. Zhang J. Ren Y. Li T. Bi J. Du Z. (2021). Luteolin Suppresses Sepsis-Induced Cold-Inducible RNA-Binding Protein Production and Lung Injury in Neonatal Mice. Shock 55 (2), 268–273. 10.1097/SHK.0000000000001624 32694396

[B62] Zhu L. Olsen R. J. Lee J. D. Porter A. R. DeLeo F. R. Musser J. M. (2017). Contribution of Secreted NADase and Streptolysin O to the Pathogenesis of Epidemic Serotype M1 Streptococcus Pyogenes Infections. Am. J. Pathol. 187 (3), 605–613. 10.1016/j.ajpath.2016.11.003 28034602 PMC5397666

